# Permeability Assessment of a High-Throughput Mucosal Platform

**DOI:** 10.3390/pharmaceutics15020380

**Published:** 2023-01-22

**Authors:** Cosmin Butnarasu, Olga Valentina Garbero, Paola Petrini, Livia Visai, Sonja Visentin

**Affiliations:** 1 Department of Molecular Biotechnology and Health Science, University of Turin, via Quarello 15, 10135 Torino, Italy; 2 Department of Chemistry, Materials and Chemical Engineering “Giulio Natta”, Politecnico di Milano, 20133 Milan, Italy; 3 Molecular Medicine Department (DMM), Centre for Health Technologies (CHT), UdR INSTM, University of Pavia, 27100 Pavia, Italy; 4 Medicina Clinica-Specialistica, UOR5 Laboratorio di Nanotecnologie, ICS Maugeri, IRCCS, 27100 Pavia, Italy

**Keywords:** mucus, permeability, drugs, PermeaPad, 3R

## Abstract

Permeability across cellular membranes is a key factor that influences absorption and distribution. Before absorption, many drugs must pass through the mucus barrier that covers all the wet surfaces of the human body. Cell-free in vitro tools currently used to evaluate permeability fail to effectively model the complexity of mucosal barriers. Here, we present an in vitro mucosal platform as a possible strategy for assessing permeability in a high-throughput setup. The PermeaPad 96-well plate was used as a permeability system and further coupled to a pathological, tridimensional mucus model. The physicochemical determinants predicting passive diffusion were determined by combining experimental and computational approaches. Drug solubility, size, and shape were found to be the critical properties governing permeability, while the charge of the drug was found to be influential on the interaction with mucus. Overall, the proposed mucosal platform could be a promising in vitro tool to model the complexity of mucosal tissues and could therefore be adopted for drug-permeability profiling.

## 1. Introduction

Drug absorption is defined as the passage of a drug into the bloodstream from the site of administration. Many factors influence this process, including a drug’s physicochemical properties, formulation, and the route of administration. Independent of the administration route, drugs must be solubilized and absorbed in order to achieve therapeutic effects.

Drug permeability across cell membranes is a critical characteristic that determines the rate and extent of human absorption and ultimately affects the bioavailability of a drug candidate. Unless intended for topical use, the crossing of semipermeable cell membranes is a necessary condition that drugs must achieve to be effective. Mechanisms of cell-membrane permeation include passive diffusion, facilitated passive diffusion, active transport, and pinocytosis [1]. The physicochemical properties of the drug (e.g., size, solubility, and lipophilicity), as well as membrane-based efflux mechanisms, can lead to poor permeability. Drugs with poor permeability are more likely to have poor absorption, distribution, metabolism, and excretion (ADME), and consequently have a lower efficacy.

Given its biological and pharmaceutical importance, approaches for the quantitative measurement of membrane permeability have been a topic of research for decades, resulting in sophisticated biomimetic systems coupled with advanced techniques [2]. Cell-based models such as the Caco-2 and Madin–Darby canine kidney (MDCK) are two of the most established systems used to measure the permeation flux of compounds and to predict in vivo absorption [3,4,5]. Such models have the benefit of considering all types of permeation mechanisms, including active transport machinery and paracellular diffusion, thus representing key features of the in vivo scenario. However, cell-based models suffer from practical limitations, such as poor standardization (e.g., gene expression), poor time and cost-effectiveness (e.g., from 4 to 21 days before use), low throughput capacity, and low reproducibility [6,7,8]. To overcome the disadvantages associated with cell- or tissue-based permeability profiling, cell-free permeation systems are gaining more interest in drug discovery and development as tools used to obtain a reliable prediction of passive absorption [9]. These systems usually consist of two compartments separated by an artificial phospholipid membrane that mimics the cellular membrane. For example, the parallel artificial membrane permeability assay (PAMPA) has been considered the state-of-the-art cell-free permeation system since Kansy et al. introduced it in 1998 [10]. Due to the fact that artificial membranes have neither active transport systems nor metabolizing enzymes, these assays are not expected to model the molecules that are actively transported. In other words, only passive diffusion can be measured using cell-free permeation systems. This is still extremely useful for classifying poorly from highly permeable drug candidates without the influence other confounding factors, especially in the early stages of drug discovery.

It is precisely in these early stages that a huge number of potential drug candidates require fast validation to assess their capacity to pass the cellular membrane. According to the FDA, almost 70% of the approved pharmaceutical products are administered via routes with mucosal barriers (e.g., oral, inhalation, and mucosal) [11]. However, due to poorly standardized protocols and a lack of validated models, none of the current in vitro, cell-free permeability systems take into account the importance of mucus in permeability assessments.

**Figure 1:**
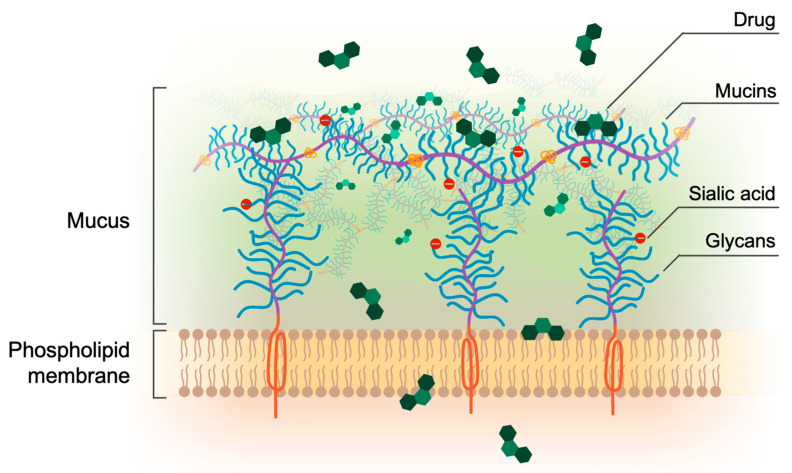
The mucosal barrier. Drugs administered by the oral, inhalation, or vaginal routes must cross the mucus layer and the cellular membrane in order to be absorbed. Due to its complex structure and chemical diversity, mucus can interact with drugs via different mechanisms and selectively filter their permeation.

Mucus is a complex, viscoelastic hydrogel lining all the wet surfaces of the human body, such as the airways, eyes, gastrointestinal tract, and vaginal tract [12,13]. Its selective permeability is governed by mucins, which are complex and heavily glycosylated proteins that form a tridimensional network around which mucus organizes (Figure 1) [14,15]. Mucus can represent a strong barrier to drugs, especially in mucus-related disorders such as cystic fibrosis and COPD [16,17,18,19]. Here, mucus dehydration and overproduction lead to stasis, which fosters a vicious cycle of chronic infection and inflammation [20,21]. Since little is known about the molecular properties that facilitate or reduce the binding of drugs to mucus, being able to measure the effect of mucus when still in the early stages of drug discovery becomes essential. Having in vitro platforms fit for this purpose could lead to the design and development of better drugs.

In this paper, we adopt a new, cell-free, high-throughput permeability model called PermeaPad to construct an in vitro mucosal platform. The recently introduced PermeaPad 96-well plate has been presented as a promising new tool for rapid-permeability profiling [22]. However, the physicochemical determinants governing its permeation mechanisms are still unclear. The main aims of this work are: (a) to selection a chemically heterogeneous dataset; (b) complete the permeability profiling of the dataset and identify the physicochemical properties ruling the permeation flux; and (c) to couple the PermeaPad 96-well plate with a cystic fibrosis mucus model and assess the impact of mucus on permeation.

Overall, this paper expects to provide insights into PermeaPad permeability mechanisms. Moreover, by setting up a new mucosal platform, we expect to provide medicinal chemists with a new tool that is useful for predicting mucosal permeability.

## 2. Experimental Section

### 2.1. Structures and Molecular Descriptors

DrugBank was used to obtain the drug simplified molecular-input line entry system (SMILES) codes [23]. DataWarrior (version 5.5.0, openmolecules.org/datawarrior), MarvinSketch (ChemAxon, ver. 22.19, www.chemaxon.com, accessed on 15 January 2023), and ADMETLab 2.0 (admetmesh.scbdd.com/) [24] were used to compute a set of 78 molecular descriptors. Computed descriptors include physicochemical descriptors, drug-likeness-related characteristics, multiple atom and ring counts, flexibility, surface area, and functional groups. Some additional descriptors were also calculated. The molecular charge and distribution coefficient at pH 7.4 were retrieved using MarvinSketch. Three-dimensional structures were obtained using Corina Demo (Molecular Networks GmbH and Altmira, LLC) and used to compute 3D-related molecular descriptors. Table S1 reports the list of all the molecular descriptors considered. The correlation matrices were computed in DataWarrior.

### 2.2. Materials

Mucin from porcine stomach (PGM Type III, bound sialic acid 0.5–1.5%, partially purified powder), calcium carbonate, sodium salt of alginic acid, D-(+)-gluconic acid δ-lactone (GDL) 99.0%, and sodium chloride were all purchased from Merck and used to create the mucus model. PermeaPad 96-well plates purchased from innoME (Espelkamp, Germany) were used for the permeability assay. An in-house Millipore system was used to generate the Millipore grade water (resistivity: 18.2 MΩ·cm at 25 °C). Acetonitrile, ammonium acetate, and dimethylsulfoxide (DMSO) of the highest available grades were purchased from Merck. The compounds used in this study were all commercially available and were obtained either from Merck or MedChemExpress (MCE). Drug solutions were freshly prepared before the permeability assay.

### 2.3. Mucus Model

The pathological mucus model produced by Bac3Gel Lda. was prepared as previously described [25,26]. Briefly, the mucus was prepared using two Luer lock syringes, mixing mucin from porcine stomach (43.8 mg/mL), alginate (21.0 mg/mL), CaCO_3_ (7.0 mg/mL), and D-(+)-glucono-δ-lactone (70.0 mg/mL), freshly prepared, in a 4:1:1:1 volume ratio, respectively. The mucin suspension was prepared in mQ water. All the other reagents were dissolved in 16.3 mg/mL of NaCl.

Next, 20 µL of the mucus mixture was pipetted into the donor compartment of the PermeaPad plate (i.e., PermeaPad + mucus, from here on). The donor plate was carefully shaken to homogeneously distribute the mucus over the entire surface of the well. This step is also useful to get rid of the air bubbles that can form during the mixing of the mucus reagents. Before running the permeation measurements, the mucus was allowed to crosslink overnight by storing the PermeaPad plate at 4 °C. The permeability of caffeine was used as a reference standard to check the reproducibility across different PermeaPad plates and different mucus batches.

### 2.4. PermeaPad Assay

The permeability assay was conducted in the absence (i.e., PermeaPad) and presence (i.e., PermeaPad + mucus) of the pathological mucus model. Stock solutions of drugs were prepared at 10 mg/mL in DMSO; working solutions were prepared by diluting the stocks within soluble concentration ranges (either 100 or 500 µM) in a 10 mM phosphate buffer (PB) (pH 7.4, 5% DMSO). The donor compartments of the PermeaPad plate were then filled with 200 µL of the drug working solution, while the acceptor compartments were filled with 400 µL of PB buffer (5% DMSO). The donor and acceptor plates were then coupled, covered with the plate lid, and incubated for 5 h. Subsequently, the aliquots from each acceptor solution were withdrawn once at 5 h, and the amount of drug passively diffused was quantified using HPLC-ESI-MS. The apparent permeability (*P_app_*) was calculated using Equation (1), derived from Fick’s law [27] for steady-state conditions.

(1) $$P_{app}=\frac{dQ/dt}{C_{0}\times A}$$

where *dQ* represents the quantity of drug expressed as moles permeated into the acceptor compartment at time *t* (18.000 s), *C*_0_ is the initial concentration in the donor compartment, and *A* is the area of the PermeaPad membrane (0.15 cm^2^).

The effect of mucus on *P_app_* was calculated as:

(2) $$\Delta \;P_{app}=\left(1-\frac{P_{app\;PermeaPad+mucus}}{P_{app\;PermeaPad}}\right)\cdot 100$$

### 2.5. Quantification

The concentration of the drug in each acceptor well was determined using a Varian HPLC equipped with a 410 autosampler and a C18 column (150 × 2 mm, 3 m, 100) and detected on a Varian 320 MS TQ mass spectrometer equipped with an electrospray ionization (ESI) source operating in positive or negative mode. The detector was used in multiple-reaction monitoring (MRM). The experimental details of each compound are reported in Table S2. Mobile phases composed of acetonitrile and water, 0.1% formic acid, or ammonium acetate (5 mM, pH 6.6), were pumped at 200 µL/min, following a gradient program. The injection volume was 5 µL. Quantifications were achieved according to a seven-point calibration curve specific to each compound.

### 2.6. Statistical Analysis

A minimum of three replicates were performed for each compound, with and without mucus. All the quantitative data are reported as the mean ± standard deviation (SD). Statistical significance was calculated by applying the Student’s *t* test; a *p* < 0.05 was considered to be statistically significant and is indicated with asterisks (i.e., * *p* < 0.05, ** *p* < 0.01, *** *p* < 0.001, **** *p* < 0.0001).

## 3. Results and Discussion

### 3.1. General Description of the Dataset

We based our study on a dataset of 35 small molecules (<1000 Da) belonging to different pharmaceutical classes. This includes antimicrobial, antiviral, anti-inflammatory, anti-tumoral, and cardiovascular drugs. These drugs were selected keeping in mind the importance of chemical variability in order to obtain an even distribution over the permeability scale. To investigate the chemical variability within our dataset, we took into account Lipinski’s rule of five (Ro5) and Veber’s rule. The first is a rule of thumb used to describe the druggability of a determinate molecule based on four descriptors: hydrogen bond donor (HBD), hydrogen bond acceptor (HBA), molecular weight (MW), and partition coefficient (logP). The second is an extension of Lipinsi’s Ro5 which predicts the druggability of a specific molecule based on the number of rotatable bonds (NRotB) and the polar surface area (TPSA).

**Figure 2:**
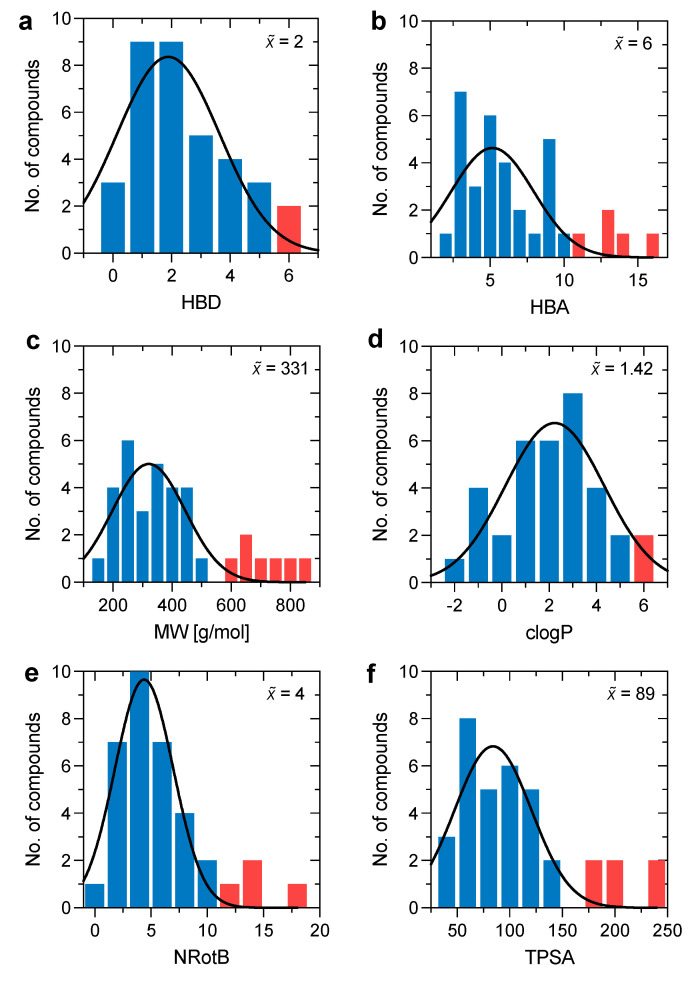
Distribution of the (**a**) hydrogen bond donors (HBD), (**b**) hydrogen bond acceptors (HBA), (**c**) molecular weight (MW), (**d**) calculated partition coefficient (clogP), (**e**) rotatable bonds (NRotB), and (**f**) topological polar surface area (TPSA) for the investigated dataset. Values that adhere to the Lipinski rule of five and Veber’s rule are colored in blue. The inset number represents the median of the dataset for each molecular descriptor.

The drugs included in the dataset cover a substantial range of the defined descriptors. Most of the compounds had values below the upper limit of Lipinski’s Ro5 (HBD ≤ 5, HBA ≤ 10, MW ≤ 500, and logP ≤ 5) and Veber’s rule (NRotB ≤ 10 and TPSA ≤ 140). However, since all the substances in the dataset are approved drugs, this should not be very surprising (Figure 2). Ro5 states that, in general, bioavailable drugs should have no more than one violation of the rule; thus, according to the median distribution within each rule, we can state that the majority of the substances we tested are bioavailable drugs. Few molecules fall over the upper cutoffs of the Lipinski and Veber rules. Despite this, these compounds are equally important for the definition of a heterogeneous chemical space.

The charge of the molecules was also considered as it is well-known that charge can play a pivotal role in permeability, especially when it is measured on non-cellular-based permeability models which lack active transports. We included charged and neutral compounds. In particular, at pH 7.4, 34% of the drugs were neutrally charged, 20% were negative, 34% were positive, and 12% were zwitterions.

### 3.2. Permeability Profiling

We measured the apparent permeability (*P_app_*) at one single timepoint (i.e., 5 h) using the PermeaPad 96-well plate. It is true that permeability measurements at multiple time points can offer a broader overview of the mechanistic aspects of passive diffusion (e.g., lag time or steady-state conditions). However, when testing moderate- to high-throughput datasets, it is preferable to ensure a high-throughput workflow. Therefore, in such cases, a single timepoint setup might be the best strategy to adopt.

The measured permeability extended from 7.4 × 10^−6^ cm/s (i.e., caffeine) to 7.9 × 10^−9^ cm/s (i.e., roflumilast) (Table 1). For only one drug (i.e., trametinib), the concentration in the acceptor compartment was below the limit of detection of the instrument. Thus, in such a case, the *P_app_* was set at 1.0 × 10^−9^ cm/s. Sink conditions were maintained throughout the experiment since the diffusion of none of the tested drugs was over 20% [28]. The permeation experiments were generally reproducible, with an average standard deviation (SD) of 27%. In particular, for 18 out of the 35 drugs, the SD was below 25%. When compared to what was previously reported [22], the SD results measured in our experimental setup are higher. This difference might be explained by the different permeability measures (i.e., one timepoint vs. multiple timepoints) and by the different quantification methods (i.e., LC-MS vs. LC-UV).

**Figure 3:**
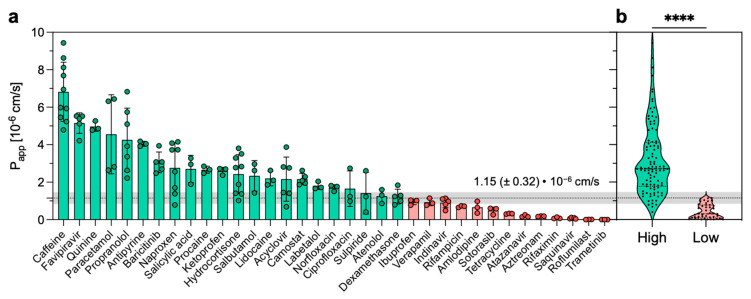
Apparent permeability (*P_app_*) of the dataset. (**a**) The dataset is classified using a binary classification (high/low) according to the *P_app_*. The threshold was set at 1.15 (±0.32) × 10^−6^ cm/s. (**b**) Student’s *t*-test was used to test the statistical significance between the high- and low-permeable groups (**** *p* < 0.0001).

Next, in an attempt to build a binary classification of the tested compounds, we divided our dataset into two permeability categories: compounds were assigned to high- or low-permeability groups depending on whether their *P_app_* was above or below 1.15 × 10^−6^ cm/s (Figure 3a). This threshold value represents the geometric mean of the entire dataset, while the error adopted (i.e., 0.32 × 10^−6^ cm/s) was the same SD (i.e., 27%) measured for the permeability values. Our permeability threshold is consistent with previously reported permeability thresholds distinguishing highly from poorly permeable compounds. For instance, on the parallel artificial membrane permeability assay (i.e., PAMPA), a cutoff value of 1.50 × 10^−6^ cm/s was used to classify high and low permeable compounds [29]. Similarly, in an attempt to provide a rank-ordering classification of discovery compounds, another study on Caco-2 cells reported < 1–2 × 10^−6^ cm/s as the cutoff to distinguish low-permeability compounds from permeable compounds [30]. The difference between the so-defined permeability groups was found to be statistically significant (Figure 3b). In addition, we also computed a principal component analysis (PCA) using Lipinski’s and Veber’s descriptors as variables and the 35 compounds tested as observations (Figure S2). Using two principal components, we explained 86.5% of the variance with good discrimination on PC1 of the high- and low-permeability drugs.

In the drug-discovery setting, the Ro5 predicts that poor absorption or permeation is more likely when there are more than five H-bond donors, more than ten H-bond acceptors, the molecular weight is greater than 500, and the calculated Log P (cLog P) is greater than five [31]. Notably, nine out of twelve compounds classified as having low-permeability violate at least one criterion of the Lipinski Ro5 (Table S3), and eight out of thirteen violate one of the two criteria of Veber’s rule (Table S4). These observations suggest that the PermeaPad model could indeed be a useful tool for predicting the bioavailability of poorly available compounds.

### 3.3. Physicochemical Descriptors Governing Permeability on PermeaPad

The PermeaPad barrier is a relatively new permeability model. It was introduced in 2013 by Di Cagno and Bauer-Brandl [32] and was only introduced as a 96-well plate in 2016 [22]. Volkova et al. reported the use of PermeaPad membranes coupled to Franz cells to construct predictive schemes of passive-diffusion permeation [33]. To date, however, the physical–chemical parameters regulating the diffusion mechanisms have never been well disclosed and, to the best of our knowledge, this is the first work where the permeability measured on the PermeaPad 96-well plate correlates to the physical–chemical properties of the drugs.

**Figure 4:**
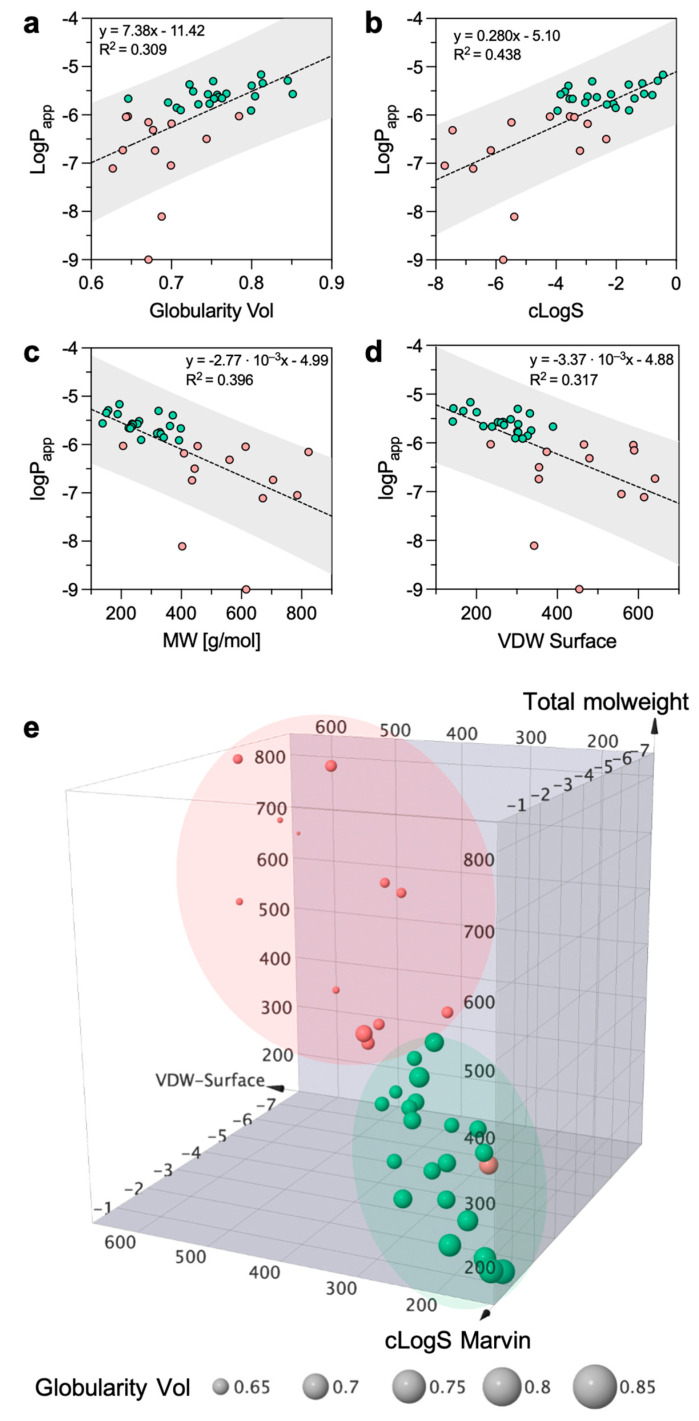
Two-dimensional plots of the logarithm of apparent permeability (*P_app_*) versus (**a**) globularity (globularity Vol), (**b**) intrinsic solubility (cLogS), (**c**) molecular weight (MW), and (**d**) the Van der Waals Surface (VDW Surface). The gray area represents the prediction bands using a 90% confidence level. (**e**) Three-dimensional plot depicting the chemical space of the dataset as defined by cLogS, MW, and VDW Surface. The marker is sized by globularity volume. A binary color code is used to distinguish between high- (green) and low- (red) permeability compounds.

To understand the influence of molecular descriptors (Table S1) on permeability data, we performed a Bravais–Pearson correlation matrix (linear correlation). To reduce the noise, we cleaned the correlation matrix by removing those molecular descriptors with a low correlation (i.e., r coefficients between −0.5 and 0.5, Table S5). The analysis showed that the best positive and negative correlations were achieved with globularity (r = 0.672), intrinsic solubility (r = 0.644), molecular weight (r = −0.681), and Van der Waals Surface (r = −0.678). To put it plainly, the more soluble and spherical the compound, the higher the *P_app_*, while the higher the molecular weight or the molecular surface, the lower the *P_app_*. Despite this, the scatter plot of log*P_app_* against globularity, cLogS, MW, and VDW Surface reveals that the proportion of variance of *P_app_* explained by the four-best correlating descriptors is low (R^2^ = 0.309, R^2^ = 0.438, R^2^ = 0.396, and R^2^ = 0.317, respectively, Figure 4a–d). However, given the data distribution, if trametinib and roflumilast are considered outliers the R^2^ would rise, revealing a much better correlation (Figure S3). Surprisingly, no significant correlation was found with lipophilicity descriptors (e.g., cLogP), even though quantitative structure–permeation relationships studies proved the predictive value of lipophilicity on membrane permeation [34]. Volkova et al. also reported a linear dependence of the permeability through the PermeaPad barrier on the solubility in 1-octanol [33].

According to the previous observations, it is evident that mainly solubility and molecular weight have good capacities to govern permeability. The relationship with globularity and Van der Waals Surface suggests that molecule shape is important in defining drug permeation. In light of these observations, we constructed a 3D scatter plot based on solubility, molecular weight, and surface. In addition, we introduced the fourth dimension by sizing each drug according to its globularity value (Figure 4e). If discriminating the high- from the low-permeability group is tricky from the individual 2D plots (Figure 4a–d), it becomes easier when looking at the 3D distribution. In fact, the two permeability groups in Figure 4e occupy different regions of the chemical space with little overlap between the two regions. Low-solubility and high-molecular-weight compounds can be discriminated from the highly soluble and low-molecular-weight structures. Furthermore, the more spherical the structure, the higher the permeability.

With non-cellular-based permeability models (e.g., PAMPA, PermeaPad, PVPA), what one sees is pure passive diffusion, mostly of uncharged species. However, it has been reported that the PermeaPad membranes also allow for the passage of compounds by paracellular diffusion; this is due to the space between the phospholipid vesicles [22,35]. If we classify the dataset into three permeability groups (instead of two), it is possible to appreciate that the high-permeability group includes mostly small, highly soluble, and globular drugs (Figure S4). Therefore, for low-molecular-weight and highly soluble compounds, it may be reasonable to assume that paracellular diffusion is the preferred mechanism of permeation in the PermeaPad. Globular shapes might be preferred to flat or linear conformations since such shapes might encounter a lower steric hindrance from the membrane. However, a more numerous dataset should be investigated to clearly identify the boundaries between the moderate- and high-permeability regions.

Overall, the computed molecular descriptors suggest that solubility, molecular size, and shape play essential roles in the permeation of PermeaPad membranes.

### 3.4. Effect of Mucus on Permeation

**Figure 5:**
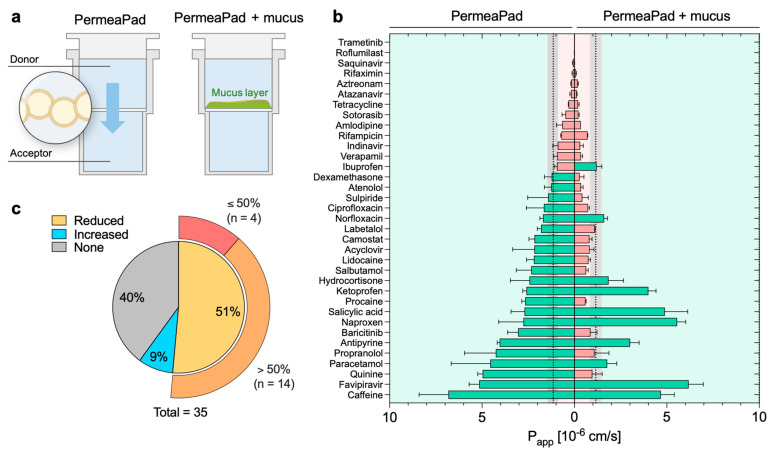
Coupling of a cystic fibrosis mucus model to the PermeaPad 96-well plate and its effect on permeability. (**a**) Graphical illustration of the different permeation setups in the absence and presence of the cystic fibrosis mucus model. (**b**) Impact of mucus on the apparent permeability of the investigated compounds. The binary color code distinguishes between high- (green) and low- (red) permeability drugs. The threshold, 1.15 (± 0.32) × 10^−6^ cm/s, is represented by the gray area. (**c**) Pie chart of the impact of mucus grouped by its overall effect on permeability compared to controls (i.e., absence of mucus). Magnification highlights the subclassification within the reduced-permeability group.

In the next step, we investigated the impact of a pathological mucus model on the permeation of the compounds tested. We used a cystic fibrosis mucus model that was adapted to the PermeaPad 96-well plate following the same approach used in our previous work [26]. The mucus was directly pipetted on top of the membrane of the donor compartment to mimic mucosal tissues (Figure 5a). The mucus model remained stable during the course of the experiment, with a minimum variation in weight and thickness (data not shown) according to what had previously been reported for the mucus model itself [25]. No visible alterations in the PermeaPad membrane were detected. The effect of mucus was evaluated by comparing the apparent permeability recorded with and without mucus (Figure S5); the effect was considered significant only when the *p*-value between the two systems was <0.05.

The mucus behaved as a dynamic and selective barrier (Figure 5b, Table 1). In particular, we observed that 40% (*n* = 14) of the drugs were not sensitive to the mucus barrier (i.e., no significant variation of *P_app_*), while 51% (*n* = 18) had a significant decrease in permeability. Among the latter group, fourteen out of eighteen compounds were strongly retained by mucus, recording a reduction of *P_app_* higher than 50% when compared to the control (i.e., without mucus). For the remaining four drugs, the decrease was below 50% (Figure 5c). A reduction in diffusivity is expected, as mucus can represent a strong barrier to the permeation of any drug acting in a mucosal environment. Key determinants of the mucus barrier are mucin glycoproteins: these can interact with drugs through a variety of low-affinity interactions (hydrophilic, hydrophobic, H-bonding, and electrostatic), reducing their overall diffusivity [17,36]. Additionally, mucus also acts as a size-exclusion filter for large particles because of the tridimensional network formed by the mucin glycoconjugates [37]. However, the mesh size of the mucus model investigated herein was estimated to be ca. 50 nm [25], making it definitely too large to impact the diffusion of small molecules.

Among the compounds tested, five drugs (i.e., ketoprofen, favipiravir, ibuprofen, naproxen, and salicylic acid) demonstrated higher permeation in the presence of mucus, although the difference was not statistically significant for salicylic acid and ibuprofen (Figure 5b,c). In our previous study [26], we proved that this phenomenon arises with a few negatively charged compounds, mostly non-steroidal anti-inflammatory drugs (NSAIDs,) as a result of complexation with the calcium present in the mucus model. After complexation, the hydrophilic–lipophilic balance of the complex shifts toward higher lipophilicity, favoring membrane permeation.

### 3.5. Physicochemical Descriptors Orchestrating the Mucus Barrier

**Figure 6:**
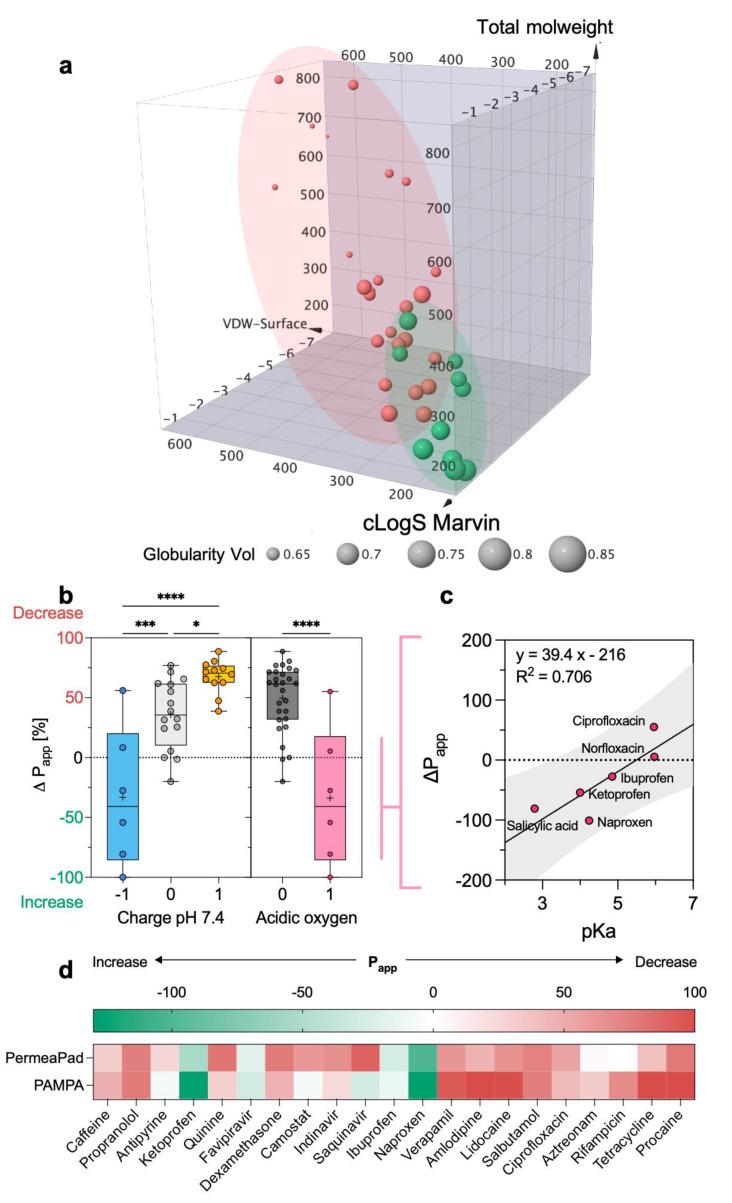
The effect of mucus and how it relates to the physicochemical properties of drugs. (**a**) Three-dimensional plot depicting the chemical space of the dataset as defined by cLogS, MW, and VDW Surface. The marker is sized by globularity volume. A binary color code is used to distinguish between high- (green) and low- (red) permeability compounds. (**b**) Box plot representation of charge at pH 7.4 and number of acidic oxygens versus the variation of permeability (Δ*P_app_*) caused by mucus. Medians are represented as black horizontal lines, while the mean is represented as a plus symbol. (**c**) The xy plot of the relationship between pKa and the variation of permeability caused by mucus. The gray area represents the prediction bands using a 90% confidence level. (**d**) Comparison between the Δ*P_app_* obtained on PermeaPad and PAMPA 96-well plates. PAMPA data are retrieved from [26] (* *p* < 0.05, *** *p* < 0.001, **** *p* < 0.0001).

After investigating how the overall drug permeation was affected by mucus, we wanted to shed light on the interplay between the drug’s physicochemical properties and the mucus barrier. Here, we investigated the molecular properties governing permeability on the new PermeaPad-mucus setup. We performed a Bravais–Pearson correlation matrix using the *P_app_* measured in the presence of mucus and the 2D and 3D structural descriptors. Again, only the highest correlation coefficients (i.e., r coefficients below −0.5 and over 0.5, Table S6) were considered. Although it presented slight variations, the correlation matrix highlighted that the main physicochemical properties involved in permeation were almost the same as those observed without mucus. In fact, the best positive and negative correlations were achieved with globularity (r = 0.691), intrinsic solubility (r = 0.513), sp^3^ hybridized carbons (r = −0.609), and Van der Waals Surface (r = −0.607). However, these similarities suggest that, even though mucus dynamically alters the kinetics of diffusivity, the limiting factor of permeability remains the crossing of the phospholipid membrane. The plot of the dataset in the same 3D chemical space previously defined (Figure 4e) shows a larger overlapping between the two permeability groups (Figure 6a), corroborating the difficulty of predicting mucus permeability.

To further define the impact of mucus on permeation, we calculated the variation in permeability (Δ*P_app_*) achieved in the presence of mucus. This new parameter allowed us to show that Δ*P_app_* is well correlated (r = 0.70) with the charge of the drug at pH 7.4 (Figure 6b). In other words, the more positive charges a molecule has, the stronger the reduction in permeability will be. This observation agrees with previous studies reporting that polycationic drugs (e.g., tobramycin, colistin, and polymyxin) strongly interact with mucins [16,38,39]. In fact, the binding of cations is highly electrostatic in nature since it involves sialic acid and sulfated sugars (e.g., galactose, N-acetyl-galactosamine, and N-acetyl-glucosamine) present in mucin’s carbohydrate chains. Regarding this last point, we also show that molecules with acidic oxygens achieve higher permeation rates in the presence of mucus (Figure 6b), possibly because they are able to form complexes with calcium. The assembly of calcium complexes with acidic oxygens is apparently dependent (R^2^ = 0.706) on the pKa of the oxygen (Figure 6c). In fact, within this limited group of drugs, four out of six drugs are pure acidic compounds (i.e., salicylic acid, ibuprofen, ketoprofen, and naproxen), while the other two are zwitterions (i.e., ciprofloxacin and norfloxacin), the latter being characterized by higher pKa values.

It is worth mentioning that the effect of mucus measured on the PermeaPad 96-well plate is in agreement with what was previously observed from PAMPA (parallel artificial membrane permeability assay) (Figure 6c) [26]. In particular, the average SD was below 30%, indicating good consistency, performance, and transferability across different permeation models.

## 4. Conclusions

Understanding and predicting the in vivo efficacy and bioavailability of potential therapeutic molecules depends heavily on permeability assessments. This process starts in early drug discovery. In order to maximize the probability of clinical success, permeability assessments should also consider the barrier effect of mucosal tissues.

In this study, we adopted computational and experimental strategies to unravel the physicochemical determinants of the permeation process on the PermeaPad 96-well plate. First, we provided a dataset of experimental permeabilities. We then showed that permeation is mainly influenced by the drug’s solubility, size, and shape. In fact, the cLogS, molecular weight, Van der Waals Surface, and globularity were found to be the best molecular descriptors to predict permeability on PermeaPad membranes. We also showed that small and highly soluble drugs use paracellular diffusion as the preferential route of permeation.

With the aim of creating a high-throughput, in vitro mucosal platform, we coupled a cystic fibrosis mucus model to the PermeaPad 96-well plate. We provided evidence that mucus can dynamically impact the permeability of drugs. We showed that the charge of the drug is an important parameter for estimating the impact of mucus. In particular, positively charged compounds will be retained by mucus barriers more than negatively charged compounds. Moreover, we also showed that drugs bearing acidic oxygens manifest an increase in permeation that is linearly dependent on the pKa of the oxygen.

Since drug development is characterized by a high rate of failure, the mucus platform described in this work could be of great translational value. It could help select the best molecules, reducing the number of ineffective drug candidates that reach preclinical trials.

Obviously, we are aware that our study considers a moderate, yet diverse, dataset that could bias results and overestimate our conclusions. Our study does not claim to exhaustively decipher the complexity of the permeability mechanism; however, it does lay a stepping stone for future permeability data interpretation.

## Figures and Tables

**Table 1:** Summary of the apparent permeabilities recorded on PermeaPad in the absence (*P_app_*) and in the presence (*P_app mucus_*) of the mucus model. Student’s *t*-test was applied to test the statistical significance between the two permeability setups (*P_app_* vs. *P_app mucus_*); significance was set at *p* < 0.05.

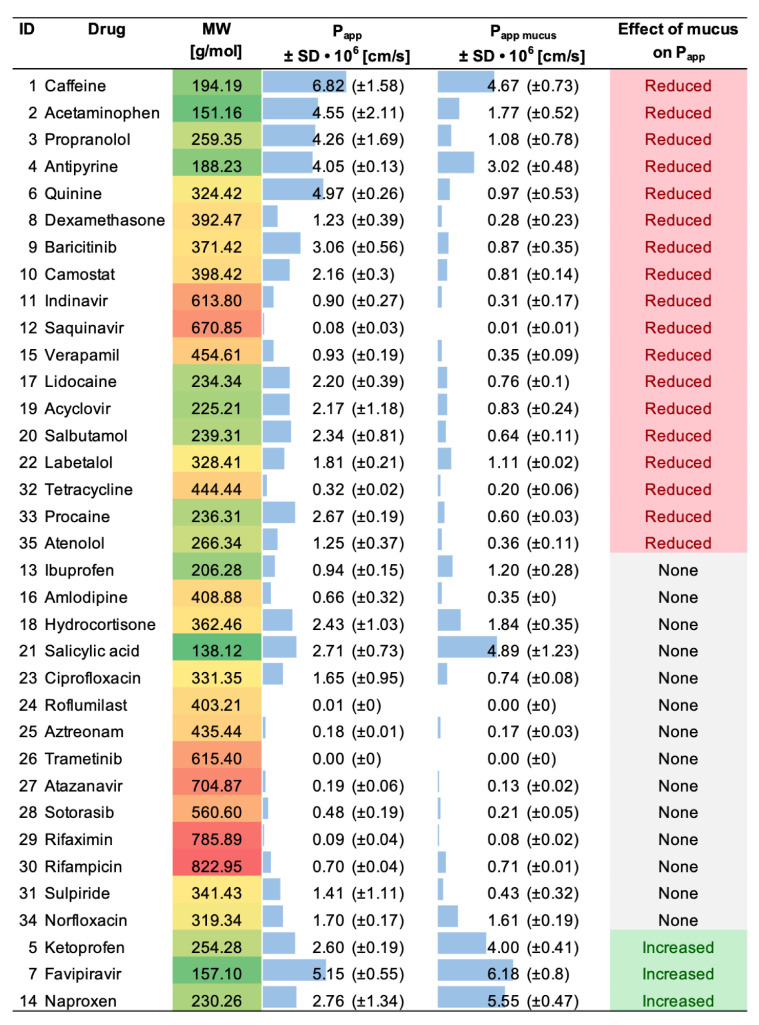

## Data Availability

The data that support the findings of this study are available from the corresponding authors upon request.

## Supplementary Materials

The following supporting information can be downloaded at: https://www.mdpi.com/article/10.3390/pharmaceutics15020380/s1. Figure S1: structures of the 35 investigated drugs. Figure S2: principal components analysis. Figure S3: 2D plots of the logarithm of apparent permeability (Papp) versus (a) globularity, (b) intrinsic solubility (cLogS), (c) molecular weight (MW), and (d) the Van der Waals Surface (VDW Surface). Figure S4: 3D plot depicting the chemical space of the dataset defined by cLogS, MW, and VDW Surface using a binary or a ternary classification system. Figure S5: comparison of the apparent permeability measured without and with mucus. Table S1: molecular descriptors dataset. Table S2: HPLC-ESI-MS analytical conditions of the investigated drugs. Table S3: Compliance of the dataset to Lipinski’s rule of 5 (Ro5). The partition coefficient (cLogP) is the average of cLogP calculated with MarvinSketch, ADMETLab 2.0, and DataWarrior. Table S4: Compliance of the dataset to Veber’s rule. Table S5: Molecular descriptors that have the strongest correlation coefficient (Bravais-Pearson) with the apparent permeability (Papp). Table S6: Molecular descriptors that have the strongest correlation coefficient (Bravais-Pearson) with the apparent permeability (Papp) measured in the presence of mucus.
