# Supplementary material for: Permeability Assessment of a High-Throughput Mucosal Platform
Source: Pharmaceutics. 2023 Jan 22;15(2):380. doi: 10.3390/pharmaceutics15020380 (PMC9966667; doi:10.3390/pharmaceutics15020380)
Supplement: Supplementary file 1 [file pharmaceutics-15-00380-s001.zip › pharmaceutics-2134898-supplementary.pdf]

Supporting Information

Permeability Assessment of a High-Throughput Mucosal Platform

Cosmin Butnarasu [1], Olga Valentina Garbero [1], Paola Petrini [2], Livia Visai [3,4] and Sonja Visentin [1,*]

[1] Department of Molecular Biotechnology and Health Science, University of Turin, via Quarello 15, 10135 Torino, Italy
[2] Department of Chemistry, Materials and Chemical Engineering "Giulio Natta", Politecnico di Milano, Milan 20133, Italy
[3] Molecular Medicine Department (DMM), Centre for Health Technologies (CHT), UdR INSTM, University of Pavia, 27100 Pavia, Italy
[4] Medicina Clinica-Specialistica, UOR5 Laboratorio di Nanotecnologie, ICS Maugeri, IRCCS, 27100 Pavia, Italy
* Correspondence: sonja.visentin@unito.it; Tel.: +39-0116708337

List of contents

Supplementary Figures

Figure S1: structures of the 35 investigated drugs.

Figure S2: principal components analysis.

Figure S3: 2D plots of the logarithm of apparent permeability (P_{app}) versus (**a**) globularity, (**b**) intrinsic solubility (cLogS), (**c**) molecular weight (MW), and (**d**) the Van der Waals Surface (VDW Surface).

Figure S4: 3D plot depicting the chemical space of the dataset defined by cLogS, MW, and VDW Surface using a binary or a ternary classification system.

Figure S5: comparison of the apparent permeability measured without and with mucus.

Supplementary Table

Table S1: molecular descriptors dataset.

Table S2: HPLC-ESI-MS analytical conditions of the investigated drugs.

Table S3: Compliance of the dataset to Lipinski's rule of 5 (Ro5). The partition coefficient (cLogP) is the average of cLogP calculated with MarvinSketch, ADMETLab 2.0, and DataWarrior.

Table S4: Compliance of the dataset to Veber's rule.

Table S5: Molecular descriptors that have the strongest correlation coefficient (Bravais-Pearson) with the apparent permeability (P_{app}).

Table S6: Molecular descriptors that have the strongest correlation coefficient (Bravais-Pearson) with the apparent permeability (P_{app}) measured in the presence of mucus.

Supplementary Figures

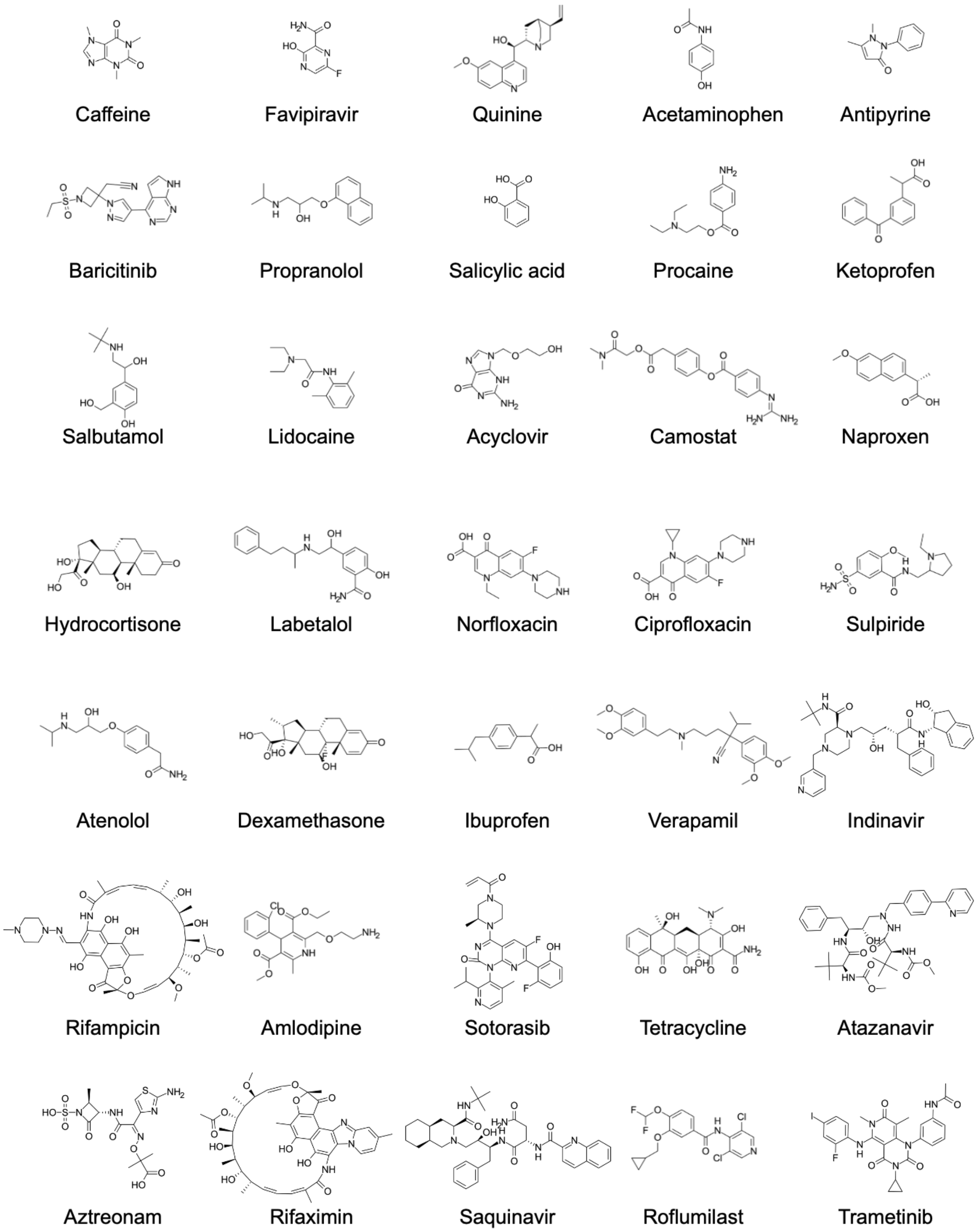

Figure S1. Molecular structures of the 35 investigated drugs.

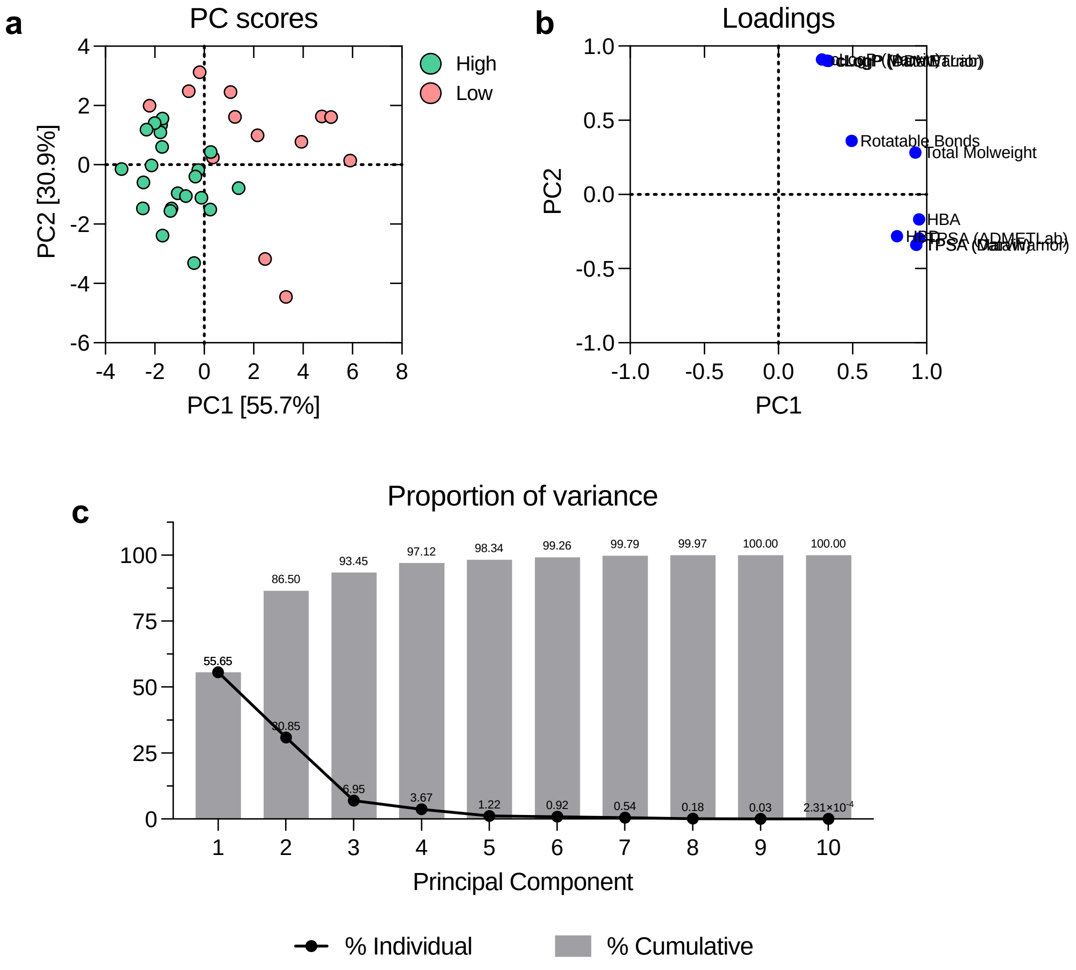

Figure S2. Principal component analysis. (**a**) Score plot of the observations along the two principal components (PC1, PC2). A binary classification color code (red, green) is used to point out the high- and low-permeable drugs. (**b**) Loading plot depicting clusters of variables. (**c**) Proportion of variance plot with the variance explained by each PC. PC1 and PC2 were selected based on parallel analysis.

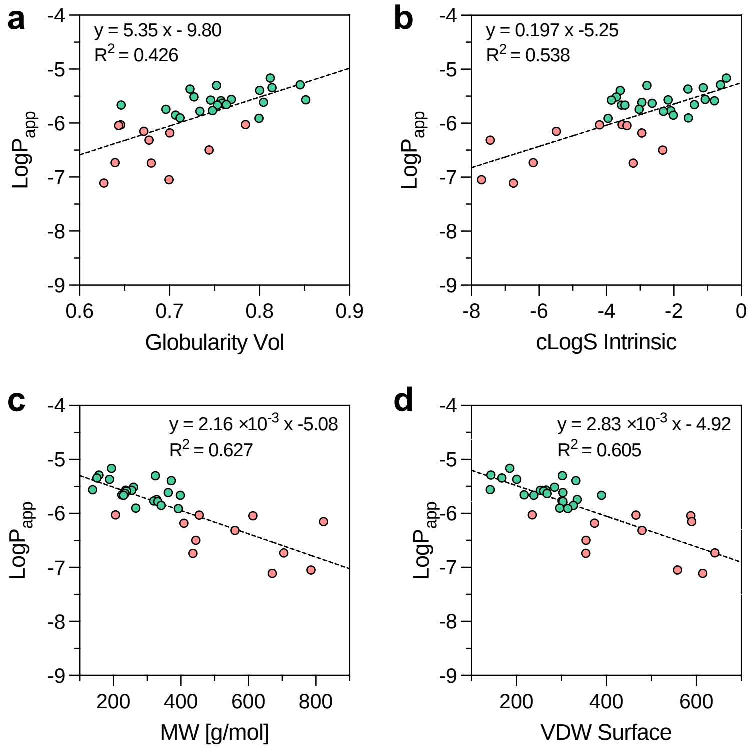

Figure S3. 2D plots of the logarithm of apparent permeability (P_{app}) versus (**a**) globularity, (**b**) intrinsic solubility (cLogS), (**c**) molecular weight (MW), and (**d**) the Van der Waals Surface (VDW Surface). The gray area represents the prediction bands using a 90% confidence level.

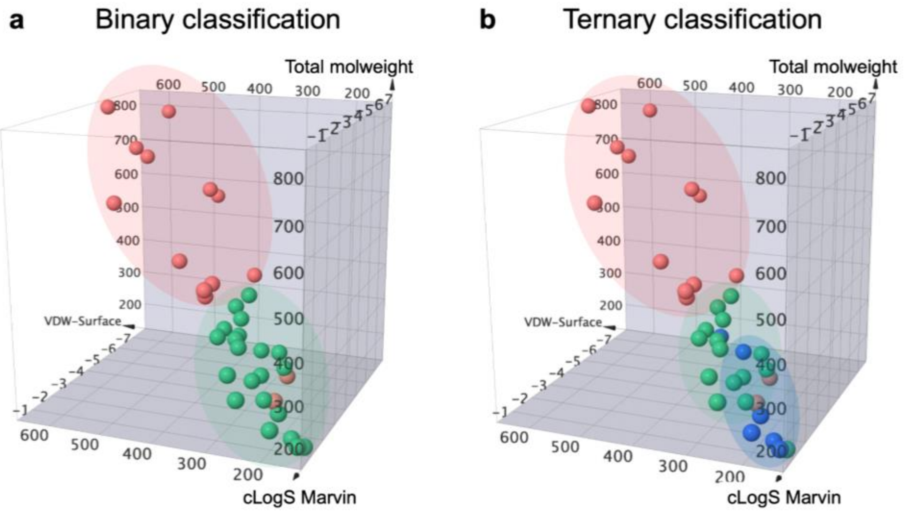

	P_{app} [· 10^{-6} cm/s]
High	≥ 1.15
Low	< 1.15

	P_{app} [· 10^{-6} cm/s]
High	≥ 4.0
Moderate	1.15 ≤ x < 4.0
Low	< 1.15

Figure S4: 3D plot depicting the chemical space of the dataset defined by cLogS, MW, and VDW Surface using a binary or a ternary classification system. The tables report the thresholds used to classify the dataset.

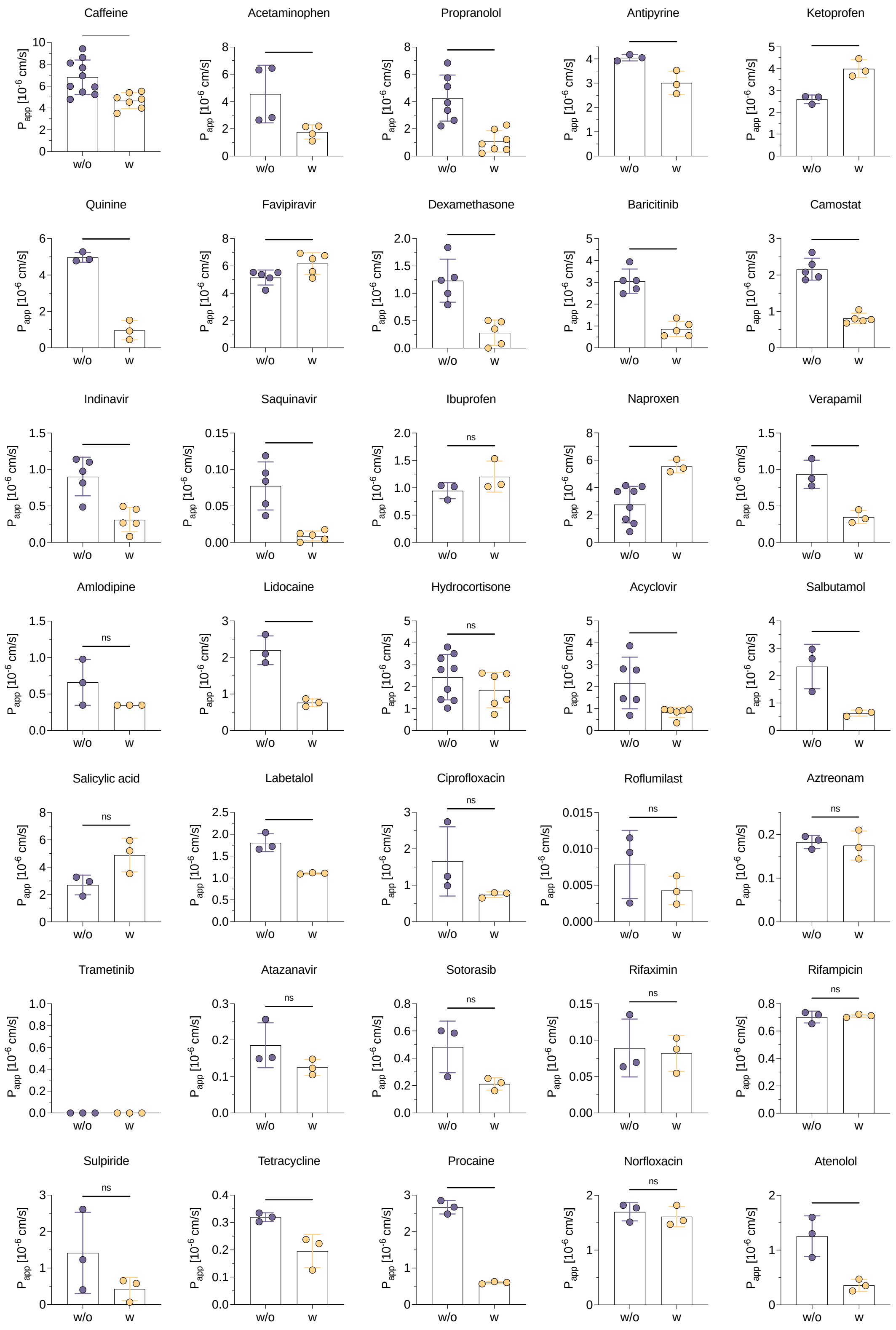

Figure S5. Comparison of the apparent permeability measured without (violet) and with (yellow) mucus. Statistical significance was calculated by applying the Student's *t* test; a $p<0.05$ was considered to be statistically significant and indicated with asterisks (i.e., *$p<0.05$, **$p<0.01$, ***$p<0.001$, ****$p<0.0001$).

Supplementary Tables

Table S1. Molecular descriptors dataset.

Marvin, ChemAxon	ADMETLab 2.0	DataWarriors, Openmolecules
Charge pH 7.4 (Nominal)	LogS	Total Molweight
Charge at pH 7.4	LogD7.4	cLogP
cLogP	LogP	cLogS
cLogD7.4	HIA	H-Acceptors
cLogS (I)	Caco-2	H-Donors
cLogS (7.4)	MDCK	Total Surface Area
Solubility mg/mL (I)	Vol	Relative PSA
Solubility mg/mL pH7.4	Dense	Polar Surface Area
logS mg/mL (I)	TPSA	Druglikeness
logS mg/mL pH7.4	nRot	Shape Index
PSA 2D	nRing	Molecular Flexibility
VDW Surface Area 3D	MaxRing	Molecular Complexity
	nHet	Fragments
	fChar	Non-H Atoms
	nRig	Non-C/H Atoms
	Flex	Metal-Atoms
	nStereo	Electronegative Atoms
	QED	Stereo Centers
	Synth	Rotatable Bonds
	Fsp3	Rings Closures
	MCE-18	Aromatic Atoms
		sp3-Atoms
		Symmetric atoms
		Small Rings
		Carbo-Rings
		Hetero-Rings
		Saturated Rings
		Non-Aromatic Rings
		Aromatic Rings
		Saturated Carbo-Rings
		Non-Aromatic Carbo-Rings
		Carbo-Aromatic Rings
		Saturated Hetero-Rings
		Non-Aromatic Hetero-Rings
		Hetero-Aromatic Rings
		Amides
		Amines
		Alkyl-Amines
		Aromatic Amines
		Aromatic Nitrogens
		Basic Nitrogens
		Acidic Oxygens
		Stereo Configuration
		Globularity SVD
		Globularity Vol
		VDW-Surface
		VDW-Volume

Table S2. HPLC-ESI-MS analytical conditions of the investigated drugs.

ID	Drug	Mobile phase	Polarity	Paren ion	Fragment ion	CE (V)
1	Caffeine	H_2O, 0.1% FA - ACN	+	195.1	137.8	-20.0
2	Acetaminophen		+	152.0	109.8	-13.0
3	Propranolol		+	259.9	116.1	-11.5
4	Antipyrine		+	189.0	76.9	-32.5
5	Ketoprofen		+	255.1	104.7	-22.0
6	Quinine		+	325.0	307.0	-18.0
7	Favipiravir		-	155.5	112.7	13.0
8	Dexamethasone		+	372.8	237.1	-20.0
9	Baricitinib		+	371.7	186.2	-34.5
10	Camostat		+	399.0	296.0	-18.0
11	Indinavir		+	614.2	421.1	-26.5
12	Saquinavir		+	671.3	570.2	-25.0
15	Verapamil		+	455.4	164.9	-21.0
16	Amlodipine		+	409.2	237.6	-14.5
17	Lidocaine		+	235.1	85.6	-14.0
18	Hydrocortisone		+	363.2	120.9	-25.5
19	Acyclovir		+	225.9	152.0	-14.0
20	Salbutamol		+	240.0	148.0	-18.5
22	Labetalol		+	329.0	162.0	-24.5
23	Ciprofloxacin		+	331.0	245.0	-20.5
24	Roflumilast		+	402.8	187.0	-26.5
25	Aztreonam		+	436.0	313.1	-15.0
26	Trametinib		+	616.4	253.9	-29.0
27	Atazanavir		+	705.8	168.0	-39.5
28	Sotorasib		+	561.4	317.0	-31.5
29	Rifaximin		+	786.8	754.6	-14.5
30	Rifampicin		+	823.6	790.9	-13.0
31	Sulpiride		+	342.1	111.9	-22.5
32	Tetracycline		+	445.2	409.2	-14.5
33	Procaine		+	237.2	99.8	-13.0
34	Norfloxacin		+	320.0	276.0	-14.5
35	Atenolol		+	267.1	144.9	-24.0
13	Ibuprofen	Ammonium acetate 5 mM, pH 6.5 - ACN	-	205.0	160.5	5.5
14	Naproxen		-	229.0	184.6	5.5
21	Salicylic acid		-	136.8	92.6	11.0

Table S3. Compliance of the dataset to Lipinski's rule of 5 (Ro5). The partition coefficient (cLogP) is the average of cLogP calculated with MarvinSketch, ADMETLab 2.0, and DataWarrior.

ID	Compound	Total Molweight [g/mol]	cLogP	H-Acceptors	H-Donors	Rule 1	Rule 2	Rule 3	Rule 4	Number of rules observed
1	Caffeine	194.19	-0.23	6	0	yes	yes	yes	yes	4
2	Acetaminophen	151.16	0.85	3	2	yes	yes	yes	yes	4
3	Propranolol	259.35	2.75	3	2	yes	yes	yes	yes	4
4	Antipyrine	188.23	0.79	3	0	yes	yes	yes	yes	4
5	Ketoprofen	254.28	3.17	3	1	yes	yes	yes	yes	4
6	Quinine	324.42	2.44	4	1	yes	yes	yes	yes	4
7	Favipiravir	157.10	-0.82	5	2	yes	yes	yes	yes	4
8	Dexamethasone	392.47	1.82	5	3	yes	yes	yes	yes	4
9	Baricitinib	371.42	0.38	9	1	yes	yes	yes	yes	4
10	Camostat	398.42	1.27	9	3	yes	yes	yes	yes	4
11	Indinavir	613.80	2.97	9	4	yes	yes	no	yes	3
12	Saquinavir	670.85	3.40	11	5	yes	no	no	yes	2
13	Ibuprofen	206.28	3.51	2	1	yes	yes	yes	yes	4
14	Naproxen	230.26	2.98	3	1	yes	yes	yes	yes	4
15	Verapamil	454.61	4.43	6	0	yes	yes	yes	yes	4
16	Amlodipine	408.88	1.86	7	2	yes	yes	yes	yes	4
17	Lidocaine	234.34	2.37	3	1	yes	yes	yes	yes	4
18	Hydrocortisone	362.46	1.59	5	3	yes	yes	yes	yes	4
19	Acyclovir	225.21	-1.68	8	3	yes	yes	yes	yes	4
20	Salbutamol	239.31	0.61	4	4	yes	yes	yes	yes	4
21	Salicylic acid	138.12	1.67	3	2	yes	yes	yes	yes	4
22	Labetalol	328.41	2.37	5	4	yes	yes	yes	yes	4
23	Ciprofloxacin	331.35	-0.26	6	2	yes	yes	yes	yes	4
24	Roflumilast	403.21	4.14	5	1	yes	yes	yes	yes	4
25	Aztreonam	435.44	-1.81	13	4	yes	no	yes	yes	3
26	Trametinib	615.40	3.79	9	2	yes	yes	no	yes	3
27	Atazanavir	704.87	4.21	13	5	yes	no	no	yes	2
28	Sotorasib	560.60	4.82	9	1	yes	yes	no	yes	3
29	Rifaximin	785.89	5.48	14	5	yes	no	no	no	1
30	Rifampicin	822.95	3.95	16	6	no	no	no	yes	1
31	Sulpiride	341.43	0.57	7	2	yes	yes	yes	yes	4
32	Tetracycline	444.44	-0.37	10	6	no	yes	yes	yes	3
33	Procaine	236.31	1.86	4	1	yes	yes	yes	yes	4
34	Norfloxacin	319.34	-0.35	6	2	yes	yes	yes	yes	4
35	Atenolol	266.34	0.34	5	3	yes	yes	yes	yes	4

Table S4. Compliance of the dataset to Veber's rule.

ID	Compound	Rotable bonds	TPSA	Rule 1	Rule 2	Number of rules observed
1	Caffeine	0	58.44	yes	yes	2
2	Acetaminophen	1	49.33	yes	yes	2
3	Propranolol	6	41.49	yes	yes	2
4	Antipyrine	1	23.55	yes	yes	2
5	Ketoprofen	4	54.37	yes	yes	2
6	Quinine	4	45.59	yes	yes	2
7	Favipiravir	1	89.1	yes	yes	2
8	Dexamethasone	2	94.83	yes	yes	2
9	Baricitinib	4	128.94	yes	yes	2
10	Camostat	9	134.81	yes	yes	2
11	Indinavir	12	118.03	no	yes	1
12	Saquinavir	13	166.75	no	no	0
13	Ibuprofen	4	37.3	yes	yes	2
14	Naproxen	3	46.53	yes	yes	2
15	Verapamil	13	63.95	no	yes	1
16	Amlodipine	10	99.88	yes	yes	2
17	Lidocaine	5	32.34	yes	yes	2
18	Hydrocortisone	2	94.83	yes	yes	2
19	Acyclovir	4	114.76	yes	yes	2
20	Salbutamol	5	72.72	yes	yes	2
21	Salicylic acid	1	57.53	yes	yes	2
22	Labetalol	8	95.58	yes	yes	2
23	Ciprofloxacin	3	72.88	yes	yes	2
24	Roflumilast	7	60.45	yes	yes	2
25	Aztreonam	6	238.2	yes	no	1
26	Trametinib	5	102.06	yes	yes	2
27	Atazanavir	18	171.22	no	no	0
28	Sotorasib	4	102.23	yes	yes	2
29	Rifaximin	3	198.38	yes	no	1
30	Rifampicin	5	220.15	yes	no	1
31	Sulpiride	6	110.11	yes	yes	2
32	Tetracycline	2	181.62	yes	no	1
33	Procaine	7	55.56	yes	yes	2
34	Norfloxacin	3	72.88	yes	yes	2
35	Atenolol	8	84.58	yes	yes	2

Table S5. Molecular descriptors that have the strongest correlation coefficient (Bravais-Pearson) with the apparent permeability (P_{app}) .

Molecular descriptor	*r* (Bravais-Pearson)	Correlation
Globularity Vol	0.672	Positive
cLogS Marvin (I)	0.644	Positive
logS mg/mL Marvin (I)	0.632	Positive
Solubility mg/mL Marvin (I)	0.618	Positive
cLogS	0.55	Positive
cLogS Marvin (7.4)	0.527	Positive
H-Donors	-0.504	Negative
Rotatable Bonds	-0.508	Negative
nRot_ADMETlab	-0.511	Negative
sp3-Atoms	-0.545	Negative
H-Acceptors	-0.547	Negative
MCE-18_ADMETlab	-0.55	Negative
PSA 2D Marvin	-0.554	Negative
Polar Surface Area	-0.555	Negative
TPSA_ADMETlab	-0.563	Negative
Electronegative Atoms	-0.596	Negative
Non-C/H Atoms	-0.596	Negative
nHet_ADMETlab	-0.596	Negative
VDW Surface Area 3D Marvin	-0.645	Negative
VDW-Volume	-0.646	Negative
Vol_ADMETlab	-0.651	Negative
Non-H Atoms	-0.652	Negative
Total Surface Area	-0.655	Negative
VDW-Surface	-0.678	Negative
Total Molweight	-0.681	Negative

Table S6. Molecular descriptors that have the strongest correlation coefficient (Bravais-Pearson) with the apparent permeability (P_{app}) measured in the presence of mucus.

Molecular descriptor	*r* (Bravais-Pearson)	Correlation
Globularity Vol	0.691	Positive
Solubility mg/mL Marvin (I)	0.513	Positive
nRot_ADMETlab	-0.508	Negative
Basic Nitrogens	-0.509	Negative
Rotatable Bonds	-0.510	Negative
sp3-Atoms	-0.534	Negative
Non-H Atoms	-0.549	Negative
Vol_ADMETlab	-0.556	Negative
VDW-Volume	-0.560	Negative
Total Surface Area	-0.564	Negative
Total Molweight	-0.569	Negative
VDW Surface Area 3D Marvin	-0.576	Negative
VDW-Surface	-0.607	Negative
Fsp3_ADMETlab	-0.609	Negative
